# Effect of mRNA/tRNA mutations on translation speed: Implications for human diseases

**DOI:** 10.1016/j.jbc.2023.105089

**Published:** 2023-07-24

**Authors:** Marcos Davyt, Nikhil Bharti, Zoya Ignatova

**Affiliations:** Institute of Biochemistry and Molecular Biology, University of Hamburg, Hamburg, Germany

**Keywords:** mRNA translation, pathogenic mutations, tRNA, mRNA, codon, tRNA abundance, SNPs

## Abstract

Recent discoveries establish tRNAs as central regulators of mRNA translation dynamics, and therefore cotranslational folding and function of the encoded protein. The tRNA pool, whose composition and abundance change in a cell- and tissue-dependent manner, is the main factor which determines mRNA translation velocity. In this review, we discuss a group of pathogenic mutations, in the coding sequences of either protein-coding genes or in tRNA genes, that alter mRNA translation dynamics. We also summarize advances in tRNA biology that have uncovered how variations in tRNA levels on account of genetic mutations affect protein folding and function, and thereby contribute to phenotypic diversity in clinical manifestations.

Ribosomes—an evolutionarily conserved molecular machine found across all domains of life—translate the information encoded in mRNA to protein. Ribosomes translate mRNA one codon at a time and sequentially add amino acids to the growing polypeptide chain in a time scale of seconds (*e.g.,* 7–15 amino acids/second for prokaryotes depending on the growth phase (1) and 3–5 amino acids/second for eukaryotes (2, 3)). The process is mediated by tRNAs, which link the nucleotide information of the mRNA with the peptide sequence of the nascent protein. Thus, tRNAs are the real “translators” of the genetic information.

More than 30 years ago, Alistair Brown and colleagues proposed in a hypothesis paper that mRNA translation should be discontinuous in order to assist the cotranslational folding of the encoded protein (4) (Fig. 1). Mounting evidence in the last 2 decades has established experimentally that the ribosomes translate mRNAs with a nonuniform velocity, with alternating segments of rapid and slow translation (5, 6, 7, 8, 9, 10). The translation velocity is under selection pressure to coordinate mRNA utilization with processes downstream of translation, such as cotranslational folding, protein expression level, interactions with other proteins and/or macromolecular machines (*e.g.*, chaperones, signal-recognition particle, enzymes posttranslationally modifying proteins), and secretion through membranes and protein assembly (11, 12, 13, 14, 15, 16, 17, 18, 19, 20, 21, 22, 23, 24, 25, 26). Several features of the mRNA can cause variations in mRNA translation speed, including codon choice (27, 28), adjacent codons or codon context (29, 30), the secondary structure of mRNAs (18), tRNA abundance and aminoacylation levels (31, 32), difficult-to-translate mRNA sequences (*e.g.*, repetitive stretches (33, 34)), or specific sequences of the nascent peptide (35, 36, 37). mRNAs exhibit a high-intrinsic propensity to form secondary structures, but these structures are rarely obstacles for the translating ribosomes (38, 39, 40). Within one species, only a few strong secondary structures have been under evolutionarily selection for specific regulatory purposes, for example, ligand-responsive riboswitches and RNA thermometers (41, 42). Similarly, the mRNA sequence itself or the nascent protein sequences influence translation of only a rather small set of proteins. Among all factors, the codon choice linked to the concentration of the cognate tRNA is the main factor that determines the translation velocity of a codon.

**Figure 1:**
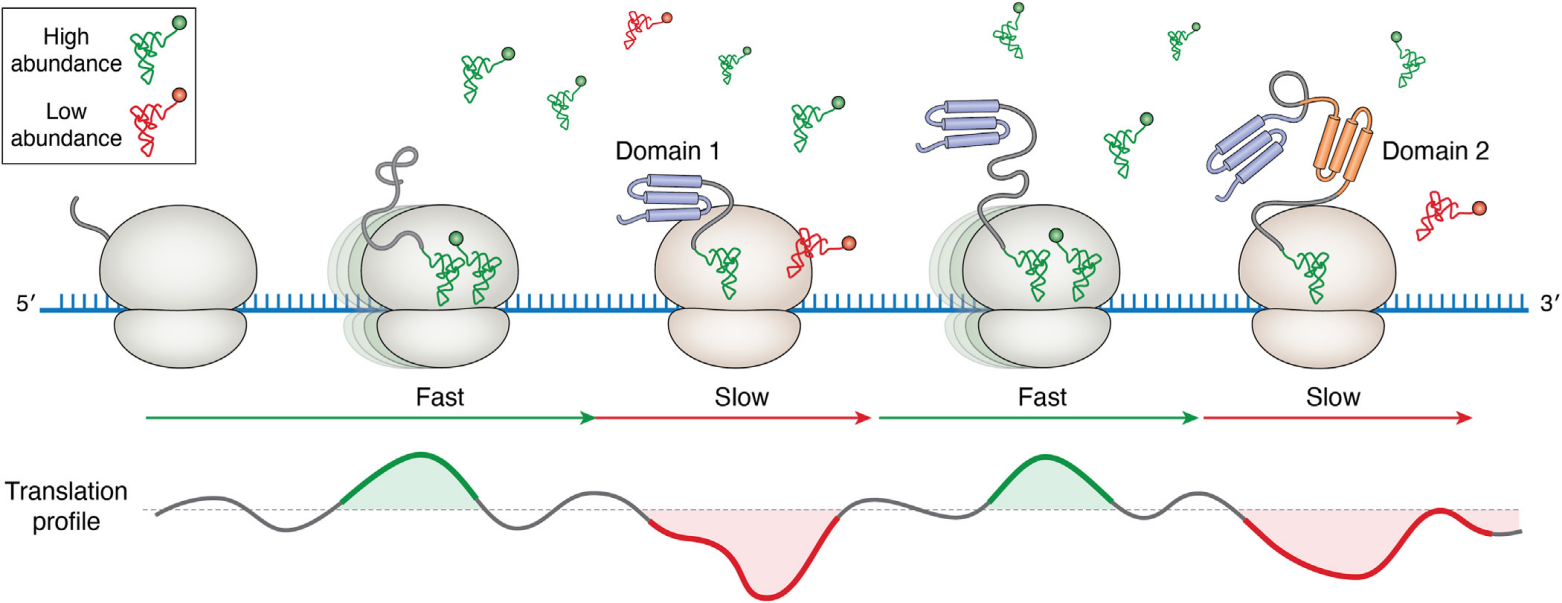
**Discontinuous translation coordinates cotranslational folding of the nascent peptide chain.** The codon translation velocity is mainly determined by the concentration of the cognate tRNA; thus, the codon sequence shapes a unique translation profile for each transcript (*discontinuous line at the bottom*). Clusters of codons pairing to low-abundance tRNAs (*red*; minima in the profile) slow the ribosome, whereas codon stretches decoded by high-abundance tRNAs (*green*) allow the ribosome to proceed with relatively fast speed. The local minima kinetically coordinate the sequential folding of single domains (domain 1, *blue*; domain 2, *orange*) in multidomain proteins.

tRNAs, charged with the corresponding amino acid by the cognate aminoacyl-tRNA synthetase (aaRS), form a complex with the elongation factor (*i.e.*, the ternary complex) and reach translating ribosomes solely by diffusion, a concentration-dependent process (43). Within an organism, the expression levels of different tRNAs vary by an order of magnitude, suggesting that the speed of decoding the slowest and fastest codon could differ by at least 10-fold. The architecture of tRNAs conforms to a narrow set of structural parameters (44, 45, 46) and sequence variations outside the anticodon compensate for the chemical diversity of the amino acid moiety (47, 48), thus enabling a similar decoding efficiency for all tRNAs (49). Posttranscriptional modifications in the anticodon also compensate for differences among tRNAs, thereby equalizing the decoding speed at the ribosome (49, 50). Except for proline codons, for which the peptide bond formation is 2-fold slower than for all other amino acids (51), the rate of decoding on the ribosome is quite uniform for all codons (52). Cumulatively, the decoding rate, which is intimately linked to the speed of three discrete processes, namely tRNA accommodation in the ribosomal A site, peptide bond formation, and translocation to the P site, is much faster than the diffusion-limited tRNA delivery to the ribosome. The decoding rate contributes approximately 5 to 10% to the overall codon translation speed (32). Under permissive growth conditions, the steady-state level of aminoacyl-tRNAs is relatively constant. Thus, the total tRNA concentration, which contributes 90 to 95% of the overall codon translation speed, serves as a good approximation for estimating a codon translation velocity (14, 20, 26) (Fig. 1).

In this review, we outline the effect of a specific group of disease-associated mutations which change the dynamics of translation at a codon and, consequently, alter the folding and function of the encoded proteins and contribute or are causally linked to pathology. Since the tRNA levels determine the speed of a codon, we propose that the concentrations of cognate tRNA pools may offer a better estimate of the effect of pathogenic mutations and that a complete understanding of this aspect could aid personalized medicine.

## Discontinuous mRNA translation is determined by codon selection linked to tRNA abundance

In protein-coding sequences, the four distinct mRNA nucleotides (A, U, C, and G) assemble into 64 unique triplets or codons, where each of 61 sense codons encode for one of the 20 canonical amino acids. The three nonsense or stop codons (UAA, UAG, and UGA) designate the end of translation. Some stop codons, combined with a specific sequence or structure and/or assisted by a dedicated translation factor, are used to incorporate modified amino acids (*e.g.*, selenocysteine, pyrrolysine, or phosphoserine) (53). In some organisms or organelles, two of the stop codons (UGA and UAG) are reassigned into sense codons (54, 55). Recent evidence highlights a unique reassignment of the UAA stop codon into a sense codon (54). However, UAA is widely accepted as the ancestral stop codon, since its reassignment into a sense codon has been avoided in virtually all organisms.

**Figure 2:**
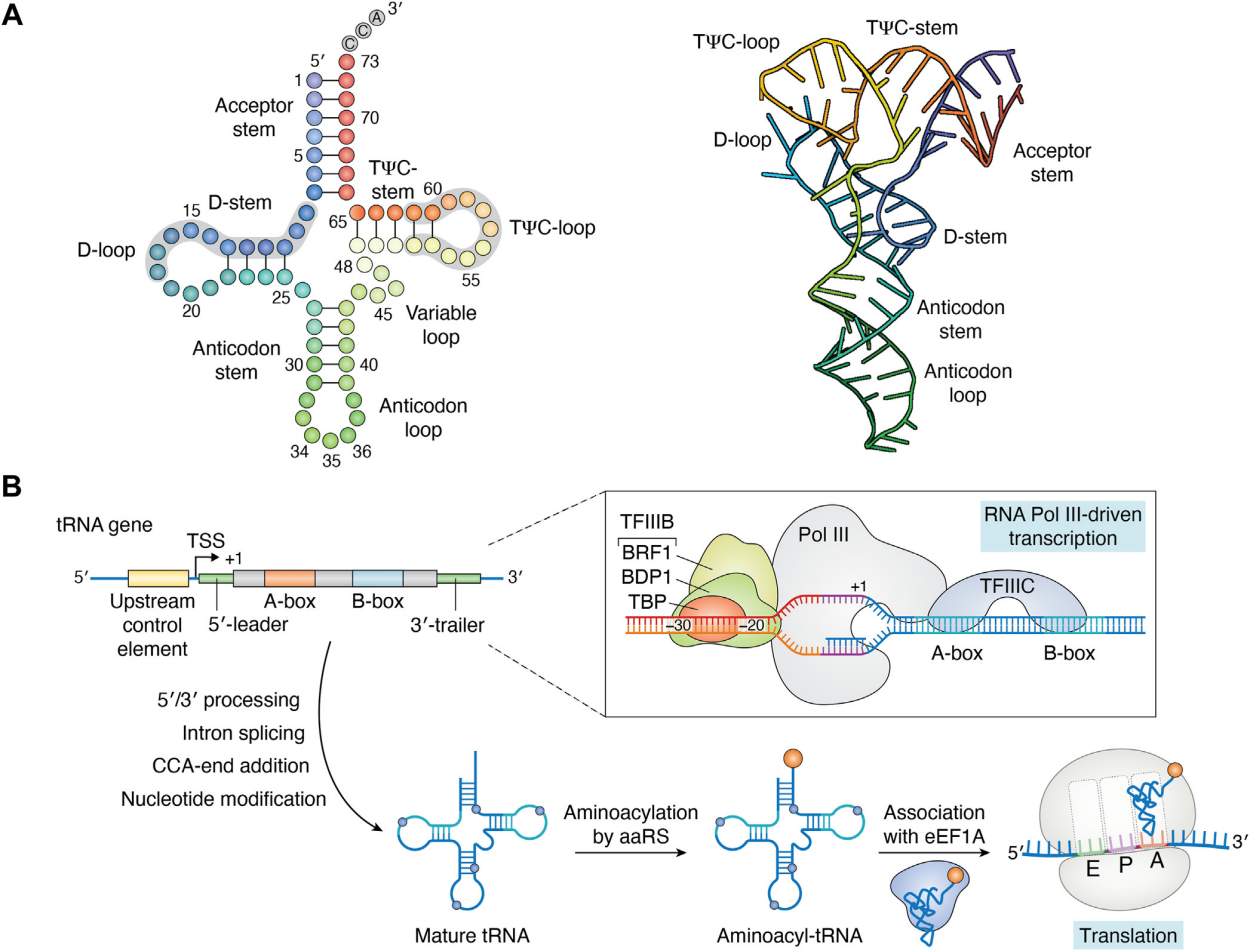
**tRNA structure, transcription, and biogenesis of eukaryotic tRNAs.***A*, “clover leaf”–shaped secondary structure (*left*) and identity elements of tRNAs (*left*). The acceptor stem is 7 bp, the D-stem is 3 to 4 bp, the TΨC-stem is 5 bp, and the anticodon stem is 5 bp. The D-loop (4–12 nt) and the variable loop, starting always at residue 44, can be 4 to 23 nt, and both introduce some variability in tRNAs length (73–90 nts). The anticodon is always numbered from 34 to 36, and the CCA tail (*white*) is always numbered from 74 to 76. Parts of the TΨC- and D-stems and loops (*gray background*) serve as internal transcription factor–binding sites. Translationally active, nuclear-encoded eukaryotic tRNAs, as well as bacterial tRNAs, adopt an L-shaped structure (*right*) as exemplified here with tRNA^Lys^(CUU) (PDB code: 7MRL). *B*, polymerase III-guided tRNA transcription is assisted by two transcription factors: TFIIIC, which binds to intragenic A-box and B-box (encoding parts of the D- and TΨC-stems and loops, *panel A*); and TFIIIB, which binds to 5′-upstream regions and constitutes of TFIIIB—BDP1 (*green*), BRF1 (*yellow*), and TBP (*orange*). +1 denotes the start of the mature tRNA. tRNA transcripts are processed in a multistep maturation process, including 5′-leader and 3′-trailer cleavage, intron splicing for some tRNAs, CCA-ends addition by the CCA-adding enzyme, and installation of posttranscriptional modifications (*blue, small balls*). Correctly processed tRNAs aminoacylated by the cognate aaRS (*orange, large ball*) form a complex with elongation factor (eEF1A) and reach the ribosomal A site by diffusion. The three tRNA-binding sites at the ribosome—A site, aminoacyl-tRNA binding; P site, peptidyl-tRNA binding, and E site, exit site—are designated. All entities in the schematic are not to scale. BRF, B-related factor; BDP, B double prime; aaRS,aminoacyl-tRNA synthetase; TBP, TATA-binding protein.

In the decoding process, tRNAs recognize sense codons by pairing to them with the anticodon sequence, a 3-nt stretch localized in the anticodon loop (Fig. 2*A*). Some codons are decoded by a dedicated tRNA whose anticodon establishes Watson–Crick interactions with all three nts in the cognate mRNA codon. Several tRNAs are modified at the first nucleotide of the anticodon loop, which enables them to pair with more than one codon through a wobble interaction with the last nucleotide of the codon (56). This arrangement reduces the total number of tRNAs required to decode all 61 sense codons. The minimum set of tRNAs to decode all sense codons is approximately 30 (57). On average, cells express approximately 35 to 55 different tRNAs. The total number of tRNAs in prokaryotes is lower than in higher eukaryotes, indicating that prokaryotes rely more frequently on wobble decoding. Bacterial ribosomes are more permissive toward wobble pairing than eukaryotic ribosomes, an observation that partly explains why plants and animals have independently evolved a greater diversity of tRNA species, leading to more precise 3-nt codon:anticodon pairing.

The genetic code is degenerate, and most of the amino acids (except Met and Trp) are encoded by two, three, four, and six codons. The average number of tRNA isoacceptors (*i.e.*, tRNAs carrying the same amino acid, but differ in the sequence of the anticodon and the tRNA body sequence) dedicated to each amino acid is relatively conserved among the three domains of life (58, 59). In humans, six amino acids (Phe, Tyr, His, Asn, Asp, and Cys) are decoded by a single-tRNA isoacceptor, while the remaining amino acids are decoded by two (Glu, Lys, Gln), three (Ile, Val, Thr, Ala, Gly, Pro), four (Ser), and five (Leu, Arg) tRNA isoacceptors. The isoacceptors carrying the same amino acid are designated as a tRNA family. Within each family, their concentration differs by 2- to 10-fold (60, 61), thus, expanding the evolutionary mechanisms to modulate translation speed by preserving the amino acid. In prokaryotes, the skewing of concentration between tRNAs in a family is much higher than in eukaryotes. For example, in *Escherichia coli (E. coli)* within the tRNA^Leu^ family, the concentration difference between the major (tRNA_CAG_^Leu^ reading CUG) and the minor isoacceptor (tRNA_UAG_^Leu^ pairing to CUA codon) is 10-fold (60). By contrast, the same isoacceptors in human HeLa cells differ 2.5-fold (62), implying that Leu codons (in HeLa cells) will be read with relatively similar velocities as opposed to a difference of 10-fold in *E. coli*. In multicellular eukaryotes, the concentration of tRNA isoacceptors varies from tissue to tissue (61, 63, 64, 65). For example, tRNA_CGU_^Thr^ is barely detectable in pulmonary tissue or bronchial cells but is present in a significantly higher concentration in heart, brain, and kidney, as well as in laboratory cell lines originating from these tissues (62). Therefore, the translation speed of identical codons would differ not only among organisms but also even between tissues and cell types.

Orthologous proteins with similar structural fold and function usually exhibit similar translation patterns, despite sharing a low-sequence homology (32). This finding suggests that translational profiles are under a selection pressure, and the coding sequence is adjusted to the tRNA pools in each species to maintain similar fidelity of cotranslational folding and function of the encoded orthologous protein. The inability to express many recombinant proteins in a soluble and physiologically active form in some heterologous hosts (66) indirectly supports the idea that translation profiles have been evolutionarily optimized to the tRNA pools of the parental cell. Adaptation of the codon usage to the tRNAome (*i.e.*, the total pool of all expressed tRNAs) of the heterologous host to reproduce the native translation profile, rather than simply replacing codons to match those that are frequently used, significantly improves recombinant expression (67, 68).

## Shaping up tRNAome composition and abundance in cells and tissues

tRNAs have a conserved cloverleaf secondary structure arranged in four stems connected by four loops: dihydrouridine-containing loop or D-loop; anticodon loop; variable or V-loop; and thymidine-, pseudouridine-, and cytidine-containing loop or TΨC-loop (69, 70). The D- and TΨC-loops establish further long-distance interactions to fold into an upside-down L-shaped conformation, with one arm comprising the acceptor stem and T-loop, and the second arm comprising the D-loop and anticodon loop (Fig. 2*A*).

Following transcription, each tRNA undergoes a series of regulated maturation events, including the removal of the 5′ leader and 3′ trailer, and intron(s) splicing (Fig. 2*B*). The prokaryotic tRNAs are encoded with their 3′-CCA termini (Fig. 2*A*, gray designated nts). In contrast, the eukaryotic tRNAs undergo a posttranscriptional addition of the 3′-terminal CCA residues by a CCA-adding enzyme, followed by a nuclear receptor–mediated export into the cytosol (71). The CCA addition and export serve as checkpoints for triaging incorrectly processed tRNAs. The 3′-CCA tail, which is a common feature of all tRNAs, serves as a platform to attach the cognate amino acid; this aminoacylation process is catalyzed by different aaRSs, each of which is specific for the 20 different canonical amino acids (72, 73).

Aside from the addition of the 3′-CCA, tRNAs undergo other extensive posttranscriptional modifications and each of them is selectively introduced by a dedicated modifying enzyme (74, 75, 76). More than 80 modifications with an average of 13 modifications per tRNA have been described for mammals (77). The average number of modifications per individual tRNA spans a large range, for example, 17 modifications on tRNA^Tyr^ from placenta (78) and three modifications for tRNA^Sec^ from HeLa cells (79). Prokaryotic and mitochondrial tRNAs (mt-tRNAs) are modified to a lesser extent, averaging eight and up to six modifications, respectively (75, 80). A comprehensive summary of cell-specific modification is presented in several reviews (69, 74, 76). Here, we highlight a number of modifications in the anticodon loop, which are indispensable for decoding fidelity. The nucleotide modifications at positions 34 and 37 of the anticodon loop stabilize AU-rich codon–anticodon pairs and aid in avoiding miscoding (50). The most common modification at the wobble position are A34 to I, which expands the base-pairing capability of tRNAs (81); 2′O-methylation of G34/C34 (Gm/Cm), which enhances codon–anticodon interactions (82), and more complex modifications, such as 5-methylaminomethyl-2-thiouridine (mcm^5^s^2^U), confer decoding bias for cognate codons (83). The identity of the nucleotide at position 37 is either A or G. N6-threonylcarbamoyladenosine (t^6^A) and N6-isopentenyladenosine (i^6^A) modifications at A37 or N1-methylguanosine (m^1^G) modification at G37 enhance accuracy of decoding by preventing frameshifting (84, 85, 86).

Among organisms, tRNAomes vary in size and composition and in multicellular organisms even among different cell types and tissues, despite the shared ancestry (19, 57, 61, 63, 64, 65, 87, 88, 89). The number of tRNA genes considerably differs across organisms, and it seems to correlate with the genome size. Unlike the protein-coding genes, which stop expanding after a certain loci number (*e.g.*, 20,000–30,000 loci per haploid eukaryotic genome), the tRNA gene copies seem to have evolved with the genome size expansion (59). Along with the large increase in tRNA copy number and gene redundancy from prokaryotes to higher eukaryotes, the anticodon repertoire and size of tRNA genes also increased (19, 59). Bacterial genomes contain between 28 and 128 tRNA genes, while eukaryotic nuclear genomes encode from 186 (*Saccharomyces pombe*) to more than 12,000 (*Danio rerio*, zebrafish) tRNA genes. In humans, there are more than 400 nuclear-encoded tRNA genes, in addition to 22 mt-tRNA genes (57, 90). Each isoacceptor is encoded by multiple genes, which are distributed in different genomic locations (57, 91). The nonidentical copies of one tRNA that bear the same anticodon but differ in sequence outside the anticodon are termed as isodecoders (92). The isodecoders could have resulted from neutral drift in larger genomes, although emerging examples assign nonredundant roles to them with tissue-specific expression patterns (65, 93, 94, 95, 96). Deleting specific isodecoders of one tRNA isoacceptor leads to different phenotypes in yeast (97). The altered tRNA body sequence of different isodecoders may modulate the efficiency of aminoacylation and interactions with ribosomes (52, 73, 98). Collectively, these studies imply that the isodecoders do not simply provide protective redundancy but may be expressed under specific conditions and/or tissues or at different levels, which in turn would affect tRNA repertoires and consequently proteome translation speed and fidelity.

Clearly, not all tRNA genes are simultaneously transcribed in every cell (96, 99). Cellular tRNA pools reflect the composition of the mRNA transcriptome (64, 100, 101). For example, the RNAomes of differentiated and proliferating cells differs and mirror the codon demand of the differentially expressed genes (64). The precise mechanisms which regulate cell- and tissue-specific tRNA levels remain elusive. In eukaryotes, tRNAs are transcribed by RNA polymerase III (pol III) facilitated by two transcription factors: TFIIIC and TFIIIB (Fig. 2*B*). Epigenetic changes and chromatin modifiers which interact with pol III determine the transcription fidelity of different tRNA genes (102). Pol III-transcribed genes, including tRNA genes, are found in comparatively nucleosome-depleted intergenic regions, arguing against a significant contribution of epigenetic alterations on tRNA expression (reviewed in (103)). More targeted tissues-specific studies, especially in the context of human pathologies, are needed to reveal the precise effect of chromatin accessibility and nucleosome dynamics. TFIIIC binds to the intragenic A-box and B-box located within the D- and TΨC-stems and loops, respectively. TFIIIB is a complex consisting of B double prime 1, B-related factor 1, and TATA-binding protein and binds upstream of the transcription start site (Fig. 2*B*). The isodecoders within one isoacceptor family display different 5′ regions (104). Different human tissues express various isoforms of B double prime 1 and B-related factor 1, which may promote expression of various sets of isodecoders and thus reflect the tissue-specific tRNA expression (104). Furthermore, nucleotides within the mature tRNA sequence but outside the TFIIIC-interacting sequences (*i.e.,* the internal A- and B-boxes) may influence tRNA transcription and expression levels. For example, the silk-producing glands of the silk-producing worm *Bombyx mori* specifically express a unique isodecoder of tRNA_AGC_^Ala^, which differs by 1 nt (*e.g.*, G40 is substituted by U) from the ubiquitously expressed tRNA_AGC_^Ala^ isodecoder (105). This finding suggests that the mechanisms which determine tissue-specific tRNA expression are likely to be complex and organism-specific.

## Pathogenic mutations associated with changes in mRNA translation velocity

**Figure 3:**
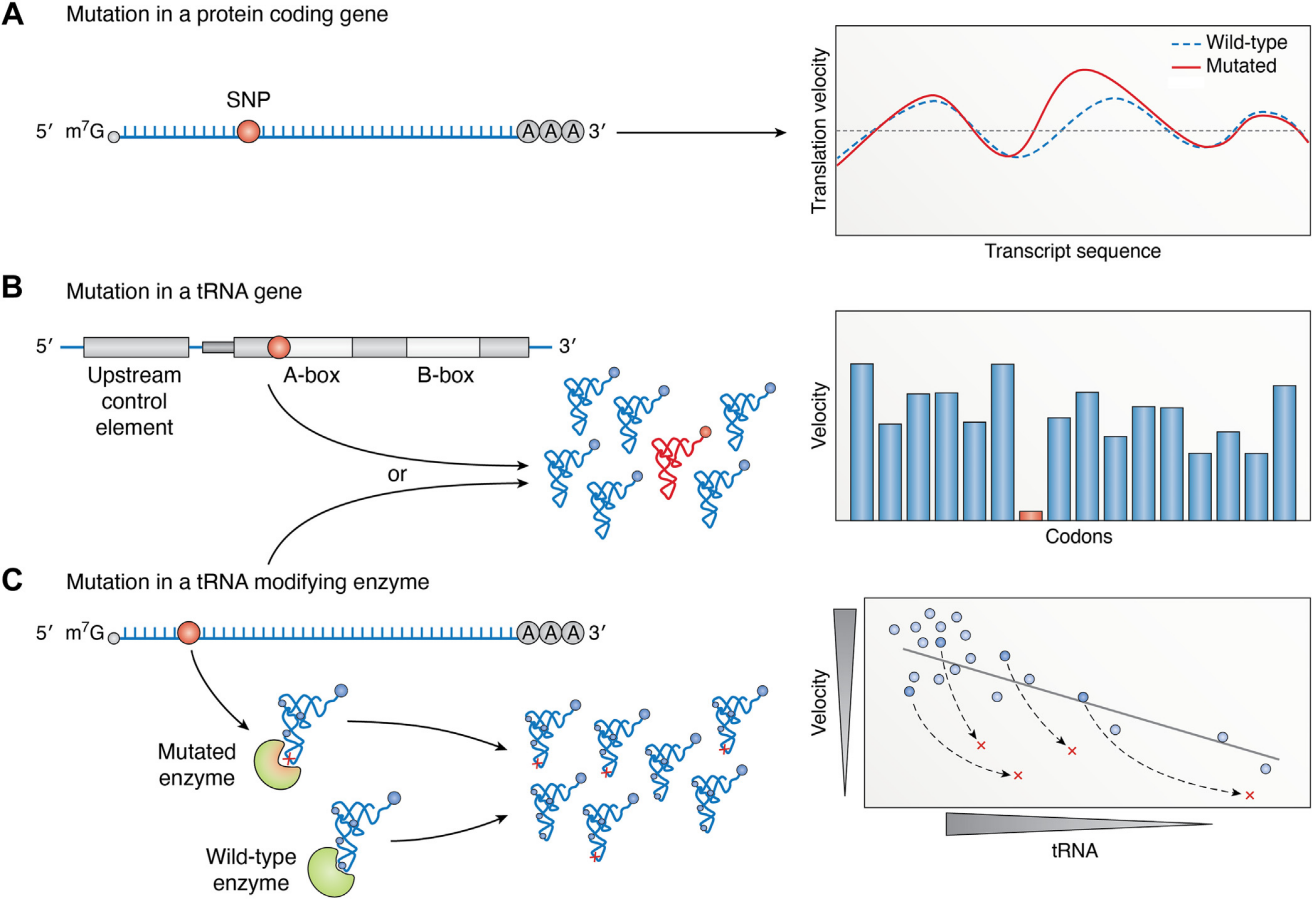
**Various types of mutations alter the speed of translation of a codon.***A*, synonymous or nonsynonymous SNPs (*red dot*) in protein-coding genes change the codon. Due to differences in the concentration of the tRNA decoding the WT and mutated codons, the translation profile of the WT transcript (*dotted blue line*) is locally affected (*red continuous line*). *B*, mutations in tRNA genes (*red dot*) alter the concentration of the affected tRNA (*red*) and consequently the translation velocity of the cognate codon (*red* on the cumulative codon velocity plot). *C*, mutations in genes encoding tRNA modifying enzymes (*red dot*) alter the conformation of the modifying enzyme (*green*) and consequently change modifications of a single tRNA isoacceptor, or in multiple tRNA isoacceptors which share the same modification; a lack of the particular modification is designated with a *red cross*. The translation velocity of the affected codon(s) decreases (*red symbols, right plot*). *Small blue dots* on tRNAs designate unaltered tRNA modifications, and *large blue dot* designate the aminoacyl-group; m7G and AAA (in *panels A* and *C*) denote the cap and polyA tail of mRNAs, respectively.

The most prevalent form of genetic variation is the single nucleotide polymorphism (SNP), known also as point mutation, by which a single base is exchanged, added, or deleted. In the coding sequence, deletions and insertions introduce a shift in the reading frame. A single-nucleotide change may not alter the ORF but can change the encoded amino acid (missense mutation or nonsynonymous SNP), introduce a stop codon and terminate protein synthesis (nonsense mutation), or cause no effect on the encoded amino acid (sSNPs). In the following sections, we outline examples of disease-associated mutations that alter translation velocity at a single codon and affect one particular protein (*i.e.,* SNPs in the cognate protein-coding gene) and mutations in tRNA genes or in genes encoding tRNA-modifying enzymes, which have more global effect and affects translation of several transcripts (Fig. 3). Across individuals, interactions among mutations known as epistasis (*i.e.,* the modulating effect of one genetic variant on another (106)) lead to variations of the spectrum of clinical manifestations and broaden the heterogeneity of disease outcomes. We discuss some examples of epistatic interactions between sSNP and nonsynonymous SNP.

### SNPs in protein-coding genes that alter codon translation speed

Both sSNP and SNP (missense) change the identity of the affected codon and may thereby alter the local speed of translation at the mutated codon. However, the evolutionarily conserved slow- or fast-translated regions are shaped by multiple codons (32, 107), which collectively determine the speed of translation of a specific mRNA segment (Fig. 1). The effect of the SNP on translation velocity would be position-specific and would depend on the buffering capacity of the flanking mRNA regions, that is, the speed of translation of the codons in the close proximity of the particular SNP. Thus, SNPs that drastically invert a codon velocity (*i.e.*, decrease or increase) are more likely to have a noticeable effect.

#### sSNPs

For a long time, have been commonly considered neutral for a protein’s structure and function. sSNPs are not randomly distributed in genomes, and they target evolutionarily conserved sites (108). Over the years, several experimental studies have shown various effects of sSNPs on mRNA structure and stability (12, 109, 110), mRNA splicing (111), protein expression, folding, stability and function (14, 28, 62, 112, 113, 114, 115, 116, 117, 118, 119), and cell fitness (120, 121).

Analysis of 33 cancer types derived from the Cancer Genome Atlas reveals a context-dependent bias of sSNPs identities (122). sSNPs that give rise to high-frequency codons and are thus likely to increase ribosomal velocity at the affected codon are more frequent in regions enriched in abundant codons and vice versa; sSNPs which are changing the codon to a low-abundance codon are more often found in slowly translated regions with predominantly low-frequency codons (122). Yet, the direct effect of the silent mutations in cancer progression and pathology remains unclear. It might be that sSNPs are not driver mutations but act in combination with other pathology mutations to dictate cancer progression and pathogenesis.

A rare synonymous mutation (c.1584G>A) in the cystic fibrosis transmembrane conductance regulator (*CFTR*) gene in a cystic fibrosis (CF) patient (Table 1), who is homozygous for this sSNP, has been recently linked as causal for pathology (123). This sSNP is located at the last position of exon 11, and the patient presents mild and rather atypical CF clinical symptoms. Typically, mutations close to the splicing junctions are associated with splicing aberrancies. This pattern was seen in patient-derived organoids where only very low levels of exon skipping and intron retention were detected (123), suggesting that these alternative splicing events cannot fully account for pathology. Reconstitution in cell culture models using the copy or complementary DNA containing the sSNP (*i.e.,* to disentangle the effects on mRNA splicing) shows alterations in the levels of mature functional CFTR. The sSNP is a GAG to GAA (Glu) codon. In bronchial epithelial cells, the concentration of tRNA_UUC_^Glu^ reading GAA is ∼50% higher than that of tRNA_CUC_^Glu^ (61, 62), suggesting that the local acceleration of translation at the mutated codon would alter the translation profile and change CFTR folding and function (123). The mild splicing aberrations caused by the sSNP appear to be offset by unexpected gains in translation speed to culminate in an atypical mild CF clinical presentation (123).

**Table 1:** Summary of all mutations discussed in this review

Mutation/gene/diseases	Affected codon or tRNA	Changes of translation speed of the affected codon
Mutations in protein-coding genes		
c.1584G>A: p.E528E/*CFTR*/CF	Glu GAG to Glu GAA	Increase by 50%
c.261G>C; p.A87A/ERα/cancer	Ala GCG to Ala GCC	5-Fold acceleration
c.135T>C)/p. S45S/*SFTPD*/COPD	Ser AGU to Ser AGC	2-Fold decrease
c.459G>A/p. V153V/hemophilia B	Val GUG to Val GUA	4-Fold decrease
c.2565T>G/p.T864T/*CFTR*/non-CF causing	Thr ACU to The ACG	Decrease by 50%
p. D579G/*CFTR*/CF	Asp GAU to Gly GGU	20-Fold decrease
p. D614G/*CFTR*/CF	Asp GAC to Gly GGC	12-Fold decrease
p. G551D/*CFTR*/CF	Gly GGU to Asp GAU	20-Fold increase
p. N1303K/*CFTR*/CF	Asn AAC to Lys AAG	No change
Mutations in tRNA genes		
m.3243A>G/mt-tRNA^Leu^(UUR)/MELAS	A14 in D-loop of mt-tRNA^Leu^(UUR)	Nearly no translation at Leu UUG
m.3271T>C/mt-tRNA^Leu^(UUR)/MELAS	U14 anticodon stem of mt-tRNA^Leu^(UUR)	Nearly no translation at Leu UUG
m.8344A>G/mt-tRNA^Lys^(UUU)/MERRF	A54 in TΨC of mt-tRNA^Lys^(UUU)	Nearly no translation at Lys AAA and Lys AAG
m.5556G>A/mt-tRNA^Trp^/OXPHOS	Variable loop of mt-tRNA^Trp^	Decrease by appr. 30% of Trp
m.10450A>G/mt-tRNA^Arg^/OXPHOS	TΨC of mt-tRNA^Arg^	Decrease by appr. 30% of Arg
m.4409T>C/mt-tRNA^Met^/mitochondrial myopathy	Between acceptor stem and D-stem of mt-tRNA^Met^	Decrease of internal AUG and initiator AUG
m.4435A>C/mt-tRNA^Met^/mitochondrial myopathy	In the anticodon loop of the mt-tRNA of mt-tRNA^Met^	N.A.
m.4450G>A/mt-tRNA^Met^/mitochondrial myopathy	Loss of the final base pair in the T-stem of mt-tRNA^Met^	N.A.
Mutations in mt-tRNA-modifying enzymes		
c.382G>A/*ADAT3/*severe intellectual disability	mt-tRNA^Ala^, mt-tRNA^Arg^, mt-tRNA^Ile^, mt-tRNA^Leu^, mt-tRNA^Pro^, mt-tRNA^Ser^, mt-tRNA^Thr^	Large decrease to nearly no translation at NN(A/C)
c.76G>C/p.A26P/*FTSJ/*nonsyndromic X-linked intellectual disability	Cm32 and GM34 of mt-tRNA^Phe^(GAA)	Large decrease at Phe UUU
Elevated levels of m^7^G mt-tRNA methyltransferase complex (METTL1 and WDR4)/hepatocellular carcinoma	mt-tRNA^Ala^(AGC), mt-tRNA^Ala^(CGC), mt-tRNA^Ala^(UGC), mt-tRNA^Arg^(UCU), mt-tRNA^Asn^(GUU), mt-tRNA^Cys^(GCA), mt-tRNA^Ile^(AAU), mt-tRNA^Lys^(CUU), mt-tRNA^Lys^(UUU), mt-tRNA^Met^(CAU), mt-tRNA^Thr^(UGU), mt-tRNA^Trp^(CCA), mt-tRNA^Tyr^(GUA), mt-tRNA^Val^(AAC), mt-tRNA^Val^(CAC), mt-tRNA^Val^(UAC)	Decrease at all codons decoded by the affected mt-tRNAs lacking m^7^G
Diverse mutations in YARS1/CMT	Sequestration of all three mt-tRNA^Gly^	Cumulative decrease at all four Gly by 75%

OXPHOS, oxidative phosphorylation; METTL, methyltransferase-like protein; N.A., not applicable; SFTPD, surfactant protein-D; WDR, WD-repeat domain.

Estrogen signaling has been linked to breast cancer progression, and the majority of breast cancers are initially dependent on estrogen (124). The estrogen receptor α (ERα)—a sterol ligand–inducible transcription factor that orchestrates pleiotropic effects in response to estrogen—bears a common sSNP (c.261G>C), which changes a rare Ala GCG codon (at position 87 of the ERα), to a major Ala GCC codon (Table 1). The tRNA levels for the relevant tissue are unknown, but considering the tRNA concentrations in related tissues (61), this sSNP would cause at least a 5-fold acceleration of the translation velocity at codon Ala 87. The sSNP-induced increase of the local translation velocity of the Ala codon alters the transcriptional activity and nuclear export of ERα (113).

Phenotypic defects linked to sSNP are also observed in chronic obstructive pulmonary disease (COPD) (125). A sSNP at codon Ser45 (c.135T>C) in surfactant protein-D is enriched in COPD patients and associated with the altered concentration of surfactant protein D in serum (125). This sSNP changes the Ser AGU codon to AGC (Table 1). Both codons are read by the same tRNA_GCU_^Ser^; however, in pulmonary tissues, the AGU codon is translated with 2-fold higher velocity (61, 62), suggesting that this subtle decrease of the decoding rate of the Ser AGT codon may over longer periods of time cumulatively affect the folding and surfactant protein-D levels in COPD patients.

Individuals with mutation c.459G>A at the Val codon (p.V153V) show mild hemophilia B (117, 126). In this case, the WT Val GUG codon of factor IX is changed to the least frequent Val GUA codon, whose cognate tRNA concentration in human cells is 4-fold lower than that of the tRNA pairing with the WT GUG codon (Table 1), suggesting a sSNP-induced translation velocity decrease at the GUA codon. The impeded translation at the mutant codon is likely affecting the folding and protein stability which could explain the observed lower extracellular protein level of factor IX (118).

Despite the plethora of examples, the effect of sSNPs on a protein’s function and cell fitness remains a matter of intense debate. Extensive libraries with more than 8000 yeast mutants—each carrying a sSNP, missense, or nonsense mutation in one of 21 endogenous genes—showed that the majority of the sSNPs caused defects in cellular fitness, which was similar to the effect of nonsynonymous SNPs (127). However, this claim was recently challenged by Kaplan and colleagues, who criticized the lack of appropriate matched controls to account for differences in the genetic background of the strains used (128). Zhang and colleagues used CRISPR-Cas9 to introduce mutations in 21 protein-coding sequences and compared the effect to a common unaltered WT yeast strain (127). However, during CRISPR-Cas9–mediated insertion of variant alleles, it is possible that unascertained fitness–altering mutations may arise and contribute to the observed fitness differences (128).

Clearly, the majority of the sSNPs are neutral with no discernible effect on the folding and function of the encoded protein. However, because of the variations of the concentration of tRNA isodecoders within one tRNA family (as outlined in the previous sections), a fraction of sSNPs may alter the speed of the affected codon and cause local alterations in the programmed translation speed. Despite the detectable mutation-driven effect on the translation speed, sSNP may remain benign. For example, a common sSNP in the human population (c.2565T>G or p.T864T) in the CFTR, that alone is not associated with CF, causes a reduction of the protein yield by 30% and a decrease of the channel activity by approximately 50% (62). This sSNP changes the WT ACU codon, which is read by the most common tRNA^Thr^ isoacceptor, to the ACG codon that is decoded by a low-abundance tRNA (62). The sSNP mutation decreases the speed of translation of the affected codon and provides a much larger kinetic window for the CFTR nascent chains to sample alternative conformations and partition in off-target pathways (20, 62, 129). For CF, the disease threshold is defined as low as 10% (130, 131), suggesting that the relation of each sSNP to pathology will strongly depend on specific disease threshold and pathology.

#### Missense mutations

Missense mutations or SNPs primarily alter the identity of the encoded amino acid. These amino acid substitutions affect folding and function of the encoded protein and are likely the major phenotypic determinant. However, the specific codon substitution may also modulate translation velocity and thereby amplify or decrease the primary amino acid substitution. Two mutations in CFTR (p. D579G and p.D614G) linked to CF pathology are associated with two identical amino acid substitutions. The p. D579G SNP changes the WT GAU (Asp) codon to GGU (Gly), and p.D614G changes the WT GAC (Asp) to GGC. Both newly mutated codons (GGU and GGC) are decoded by minor tRNAs and the tRNA concentration changes induced by p. D579G and p.D614G are 20-fold and 12-fold, respectively (132). Thus, in addition to the amino acid alteration, both missense SNPs would decrease the velocity of the affected codons. In another CF-causing mutation (p.G551D), the tRNA_GUC_^Asp^, which decodes the WT codon is 20-fold higher than tRNA_UCC_^Gly^ that pairs with the mutant GAU codon, thus implying a speed deceleration at the mutated codon. By contrast, the mutation (p.N1303K) leads to an exchange of Asn AAC codon to Lys AAG. Because the concentration of the cognate Asn and Lys tRNAs is similar, both codons will be translated at a similar velocity (132). It is likely that SNPs that alter translation velocity modulate the primary defect from the amino acid substitution and may have crucial implications in treatment with modulators that improve CFTR folding. For example, p.N1303K mutation is refractory to treatment with Food and Drug Administration/European Medicines Agency-approved drug, Trikafta/Kaftrio (elexacaftor/tezacaftor/ivacaftor), whereas G551D, D579G, and D614G mutations are responsive and their treatment with Trikafta/Kaftrio has been approved ((133) and https://www.cff.org/sites/default/files/2022-02/Trikafta-Approved-Mutations.pdf). Indisputably, the primary effect on folding, stability, and function of the encoded protein is due to the amino acid substitution. However, it is possible that translation speed alterations at the mutated codon might be beneficial by providing a longer time window for the drug to modulate the cotranslational folding of the mutated CFTR.

#### Epistasis

**Figure 4:**
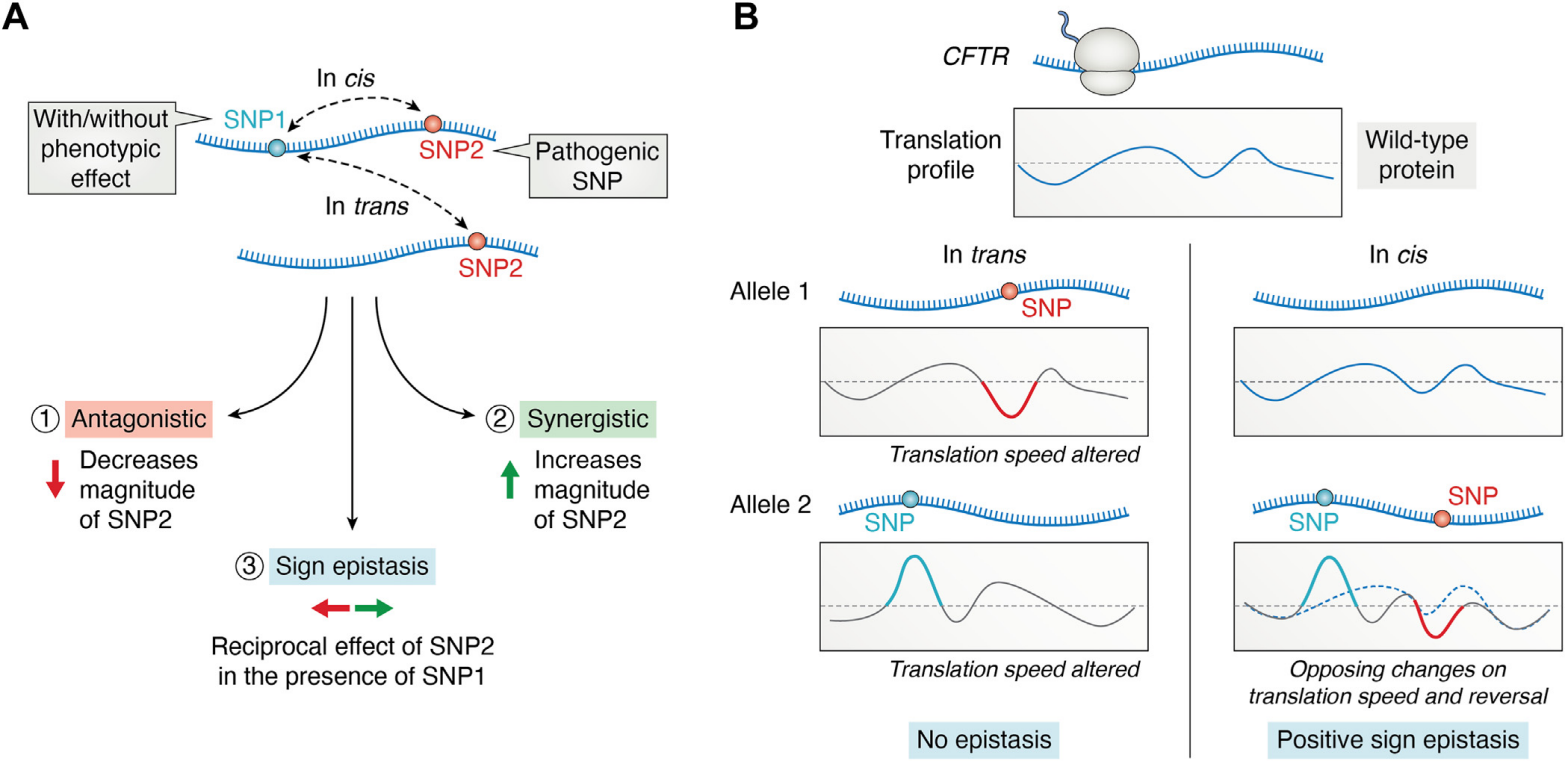
**Epistatic interactionsbetween mutations.***A*, epistatic interactions between two mutations (SNP1 [*blue dot*] and SNP2 [*red dot*], both on one allele [*cis*] or on two different alleles [*trans*]) decrease the magnitude of the mutational effect (antagonistic effect 1), increase the magnitude of the mutational effect (synergistic effect 2), or completely change the sign of the effect (3, positive, or negative sign-changing epistasis). SNP1 and SNP2 can both be missense mutations or sSNPs or a combination thereof. *B*, example of positive sign epistasis described in *CFTR*. Both the c.2562T>G sSNP (*red*) or the disease-causing missense SNP (*blue*) alter the translation speed at the affected codons and decrease CFTR channel function. There is no epistasis when both mutations are on different alleles (*trans*). In *cis*, both mutations synergize (*continuous line* with color coded segments impacted by the corresponding mutation) into a positive sign epistasis and alter the translation profile (*dotted line*) to more closely resemble that of the WT CFTR (*blue continuous line, upper profile*). CFTR, cystic fibrosis transmembrane conductance regulator; sSNP, synonymous SNP.

Epistatic interactions can alter the magnitude of the mutational effect (antagonistic or synergistic epistasis) or completely change the sign of the effect (positive or negative sign–changing epistasis) (Fig. 4*A*). By definition, if two mutations lead to a greater fitness phenotype or improvement in protein’s function than that observed with each mutation alone, it is referred to as positive epistasis (134, 135, 136). Conversely, poorer fitness phenotype is a result of negative epistasis where the effect of two mutations together results in a greater fitness decrease or drop dampening of protein’s function than each single mutation. Independently of the sign of the epistasis, when the effect of two mutations is amplified, the interaction between them is referred to as synergistic epistasis and vice versa by the antagonistic epistasis when the effect of both mutations together is smaller than the effects of the two single mutations. Therefore, for adverse phenotypes, negative epistasis is also synergistic and the positive epistasis would be antagonistic. In turn, for combined mutations improving the phenotype, the positive epistasis is synergistic and negative antagonistic (134, 135, 136).

The most prevalent sSNP in *CFTR* (c.2565T>G or p.T864T) does not cause disease even though it decreases the speed of translation at the Thr 845 (62). When this sSNP is on the same allele with some CF disease–causing missense mutations (*e.g.,* p.G85E, p.G551D, p.D579G, p.D614G), it exhibits strong positive epistasis and enhances CFTR expression and activity (Fig. 4*B* and (132)). The c.2565T>G sSNP affects only missense mutations in *cis*, which are also linked to alterations of the translation velocity at the affected codon (Fig. 4*B*). Changes of the codon which is affected by the missense SNP to an alternative codon that does not change translation speed cancelled out the positive epistasis effect (132), suggesting that the synergistic alteration of the ribosomal velocity at both missense SNP and sSNP is the underlying mechanism. The Thr 854 codon is located at a strategic position in the CFTR structure, that is, at a point crucial for establishing domain–domain interactions (137). In the context of the missense variant in *cis*, the c.2562T>G sSNP–induced reduction of translational velocity may provide an additional time window for establishing crucial domain–domain interactions that are otherwise perturbed by each individual missense mutation.

Such epistatic interactions highlight the importance of complete gene sequencing for accurate diagnosis. Unraveling epistatic interactions between mutations will help better understand the disease heterogeneity among individuals, and the ability to predict epistatic effect will be crucial in designing effective individualized treatments.

### Mutations that alter tRNA abundance and consequently codon translation velocity

Mutations in tRNA genes or in genes encoding enzymes involved in tRNA biogenesis (*e.g.,* aaRSs and tRNA-modification enzymes) will alter the concentration of functional, translationally competent tRNAs and consequently influence codon velocity. Unlike mutations in protein-coding genes, which cause an alteration of the translation velocity of the affected codon, this group of mutations would affect all transcripts with a specific codon (Fig. 3, *B* and *C*). Here, we highlight examples in which the pathogenic mutation alters translation speed. The underlying pathology mechanism of mutations in tRNA genes and proteins involved in tRNA biogenesis differ, and a much deeper overview in the diversity of the effects are summarized in several excellent reviews (69, 76, 138, 139, 140).

#### Mutations in tRNA genes

Two devastating pathologies are connected to mutations in mt-tRNA genes, that is, mitochondrial encephalopathy, lactic acidosis and stroke-like episodes (MELAS), and myoclonus epilepsy associated with ragged-red fibers (MERRF) (141, 142). The most common mutations in MELAS (m.3243A>G and m.3271T>C) are located in the D-loop (A14), and in the anticodon stem (U40) of mt-tRNA_UUR_^Leu^, respectively (143, 144). The most prevalent m.8344A>G in MERRF replaces A54 in the TΨC of mt-tRNA_UUU_^Lys^ (145). Although all mutations are located in distinct parts of the tRNA body, they share a common functional signature (Table 1): they alter the modification at U34 in both mt-tRNA_UUR_^Leu^ and mt-tRNA_UUU_^Lys^, which then destabilizes the U:G wobble interaction and alters fidelity of decoding and translation speed at a cognate wobble codon (143, 146). In both MERRF and MELAS, the level of the aminoacyl-tRNA markedly decreases (147, 148), suggesting a translation-speed–related defect. However, MERRF-linked mutation is more detrimental for the cell than the mutations associated with MELAS (149). m.8344A>G renders mt-tRNA_UUU_^Lys^ unable to translate both Lys AAA and AAG codons, while m.3243A>G or m.3271T>C influences the decoding of Leu UUG but not Leu UUA codon.

The pathology of oxidative phosphorylation system deficiencies is associated with mutations in two mt-tRNAs: m.5556G>A is located in the variable loop of mt-tRNA^Trp^ and m.10450A>G is located in the TΨC-stem of mt-tRNA^Arg^ (150). Analysis of patient-derived fibroblasts show that both mutations lowered (by 29%) the steady-state levels of the mutant mt-tRNAs (150). Such concentration changes would only marginally lower the speed of translation of the cognate codons (*i.e.,* also by approximately 30%; Table 1). However, for tissues or organelles (mitochondria) with high-translation efficiency, even small changes would be consequential over time. Indeed, the disease is lethal in early childhood with rapidly progressing symptoms.

Mutations in the gene of human mt-tRNA^Met^ (m. 4409T>C, m.4435A>C, m.4450G>A) are linked to mitochondrial myopathy, leading to muscular dystrophy and exercise intolerance. *In vitro* structural probing of mt-tRNA^Met^ carrying the m. 4409T>C mutation (*i.e.,* located in the unpaired region between the acceptor stem and D-stem; Table 1) reveals large structural alterations (151). Only a small fraction of the synthesized mt-tRNA^Met^ can be aminoacylated, which leads to a drastic reduction of the level of methionyl-mt-tRNA^Met^ and consequently a slower speed of translation of internal AUG codons (151). Mitochondria contain a single mt-tRNA^Met^ gene to decode internal and initiating AUG codons. The fraction of mt-tRNA^Met^ that is designated for initiation is additionally formylated (151). The mutation-associated structural alterations decrease the proportion of the formylated initiator mt-tRNA^Met^. Therefore, in addition to the global decrease of methionyl-mt-tRNA^Met^, the initiation frequency is decreased thereby affecting virtually all mitochondrial transcripts (Fig. 3*B*).

#### Mutations in tRNA processing enzymes that alter tRNA abundance

A-to-I editing at position 34 is a posttranscriptional process generating diversity in decoding, wherein I at wobble position 34 (I34) translates codons with U, C, or A as third base (152). In humans, this modification is incorporated into precursor tRNAs or during tRNA maturation and is catalyzed by the heterodimeric enzyme adenosine deaminase tRNA-specific 2 and 3 (ADAT2 and ADAT3) (153, 154, 155). This modification is indispensable for several tRNA isoacceptors, which decode the NN(A/C) codons for eight amino acids (*e.g.,* Ala, Arg, Ile, Leu, Pro, Ser, and Thr). A missense mutation in *ADAT3* (c.382G>A), identified by exome sequencing, is linked to severe intellectual disability (156, 157). Recently, a second mutation in *ADAT3*—an 8 bp duplication (c.99_106dupGAGCCCGG)—was reported in a patient with mild intellectual disability (158). Although the levels of A-to-I editing have not been directly analyzed in patients, a knockdown of the human *ADAT* to mimic the effect of pathogenic mutations decreases the I34 modification levels of all potential tRNA substrates (154). For the affected NN(A/C) codons, this scenario would be equal to a partial to complete lack of the cognate tRNAs and large decrease of the speed of several codons (Fig. 3*C* and Table 1).

The human FTSJ, a 2′-O-methyltransferase, introduces Cm32 and Gm34 in the cytosolic tRNA_GAA_^Phe^ that decodes both UUU and UUC codons. The FTSJ-associated 2′O-methylations are interdependent and hierarchical *in vivo* (159). A missense mutation (p. A26P) in the *FTSJ* gene leads to a lack of Gm34 and Cm32 modifications in tRNA_GAA_^Phe^ and is linked to nonsyndromic X-linked intellectual disability (82). The Cm32 and Gm34 modifications play a crucial role in decoding UUU codons, which are particularly enriched in genes expressed in the brain (159). Although the effect of tRNA_GAA_^Phe^ lacking Cm32 or Gm34 modifications has not been directly measured in patients, in *FTSJ1* KO HEK293T expressing tRNA_GAA_^Phe^ lacking Gm34 modification shows altered translation velocity at UUU codons but not at UUC codons (159), affecting the expression of all transcripts containing a UUU codon (Fig. 3*B* and Table 1) Thus, the pathogenic missense (p.A26P) mutation affects translation velocity in a manner similar to m.3243A>G or m.3271T>C-driven alterations in mt-tRNA_UUR_^Leu^ in MELAS.

tRNA modifications as a potent regulator of tRNA abundance play an essential role in tumorigenesis and tumor progression. The levels of m^7^G tRNA methyltransferase complex components (methyltransferase-like protein-1 and WD repeat domain 4 (WDR4)) are elevated in hepatocellular carcinoma and associated with a poor clinical outcome. Functionally, higher methyltransferase-like protein-1/WDR4 levels enhance the levels of m^7^G-modified tRNAs (*e.g.,* tRNA_AGC_^Ala^, tRNA_CGC_^Ala^, tRNA_UGC_^Ala^, tRNA_UCU_^Arg^, tRNA_GUU_^Asn^, tRNA_GCA_^Cys^, tRNA_AAU_^Ile^, tRNA_CUU_^Lys^, tRNA_UUU_^Lys^, tRNA_CAU_^Met^, tRNA_UGU_^Thr^, tRNA_CCA_^Trp^, tRNA_GUA_^Tyr^, tRNA_AAC_^Val^, tRNA_CAC_^Val^, and tRNA_UAC_^Val^), which promote hepatocellular carcinoma proliferation, migration, and invasion (160). m^7^G-modified tRNAs regulate the translation of oncogenic transcripts, including cell-cycle and epidermal growth factor receptor pathway genes (161). The effect depends on translation, as reduced m^7^G modification on tRNAs results in ribosome stalling and decrease in the translation velocity of codons decoded by m^7^G-modified tRNAs (161), thus, affecting a plethora of transcripts (Fig. 3*C* and Table 1).

Ten monoallelic diseases arising from autosomal dominant variants in seven genes encoding cytosolic aaRSs have been identified. The most common condition, the Charcot-Marie-Tooth disease (CMT), is associated with dominant, monoallelic mutations in six *aaRSs* genes (*e.g.,* *YARS1*, *MARS1*, *KARS1*, *WARS1*, *AARS1*, *GARS1*, and *HARS1*). CMT is a hereditary neurological disorder affecting the peripheral nervous system and manifests as progressive loss of motor and sensory functions (162, 163, 164). Establishing a molecular mechanism to explain the common pathology has been difficult, as many pathogenic mutations do not alter the aminoacylation activity. Recent studies with pathogenic GARS variants provide a new mechanism to explain the common disease pathology for the CMT-GlyRS variants (165). In the aminoacylation cycle, CMT-associated GARS variants bind tRNAs^Gly^ but fail to release them, thereby transiently depleting the cellular glycyl-tRNA^Gly^ pool. Ribosome profiling (also called Ribo-seq), which captures translating ribosomes at cell-wide scale and provides a means to measure translation speed at single codon or across transcripts (166), has been used to quantify the translation velocity across transcripts. The transient sequestration of the glycyl-tRNA^Gly^ by the mutant CMT-GARS causes a reduction in the translation velocity of all four Gly codons (Table 1) (165). This transient ribosome stalling at Gly codons chronically activates the integrated stress response in the affected motor neurons through the sensor general control nonderepressible 2 kinase (165, 167).

## Predicting the effect of pathogenic mutations on translation velocity

The increasing number of examples of mutation (single or epistatic)-driven translation speed alterations, which in turn modulate disease heterogeneity and severity, motivates development of methods to predict this effect. Since tRNA abundance is the major determinant of the translation velocity of a codon, quantification of tRNAomes could serve as a good predictor of the effect of a mutation. Determining tRNA levels has been notoriously difficult because tRNA sequences are short, heavily modified, and contain tight secondary structures. A wealth of data suggests that the tRNA gene copy numbers can be used as a proxy for cellular tRNA concentration in unicellular organisms (*e.g., Saccharomyces cerevisiae*) (27). In higher eukaryotes, however, even considering species-specific covalent modifications (81), the correlation between genomic copy number and tRNA concentration remains poor. Microarray technology was the first approach adopted for measuring tRNA abundance and charging levels, using tDNA probes complementary to full-length tRNAs (63, 168, 169). A limitation of this method is that the temperature used for hybridization of tRNAs to their cognate probes compromises the resolution of the microarrays, so that isodecoders and isoacceptors with sequence differences of fewer than 8 nts cannot be distinguished. Yet, the tRNA microarrays allowed unprecedented insights into the dynamics of tRNA pools across species (168, 169, 170, 171, 172, 173, 174, 175, 176, 177) and among tissues in higher eukaryotes (61, 63). The advent of deep-sequencing technologies tailored to tRNA-specific features enables more accurate quantification of dynamic tRNA populations. Examples for such technological developments are mim-tRNAseq (178), QuantM-seq (89), YAMAT-seq (179), hydro-tRNAseq (180), DM-tRNA-seq (181) PANDORA-seq (182) and direct tRNA sequencing using nanopore technology (183, 184).

**Figure 5:**
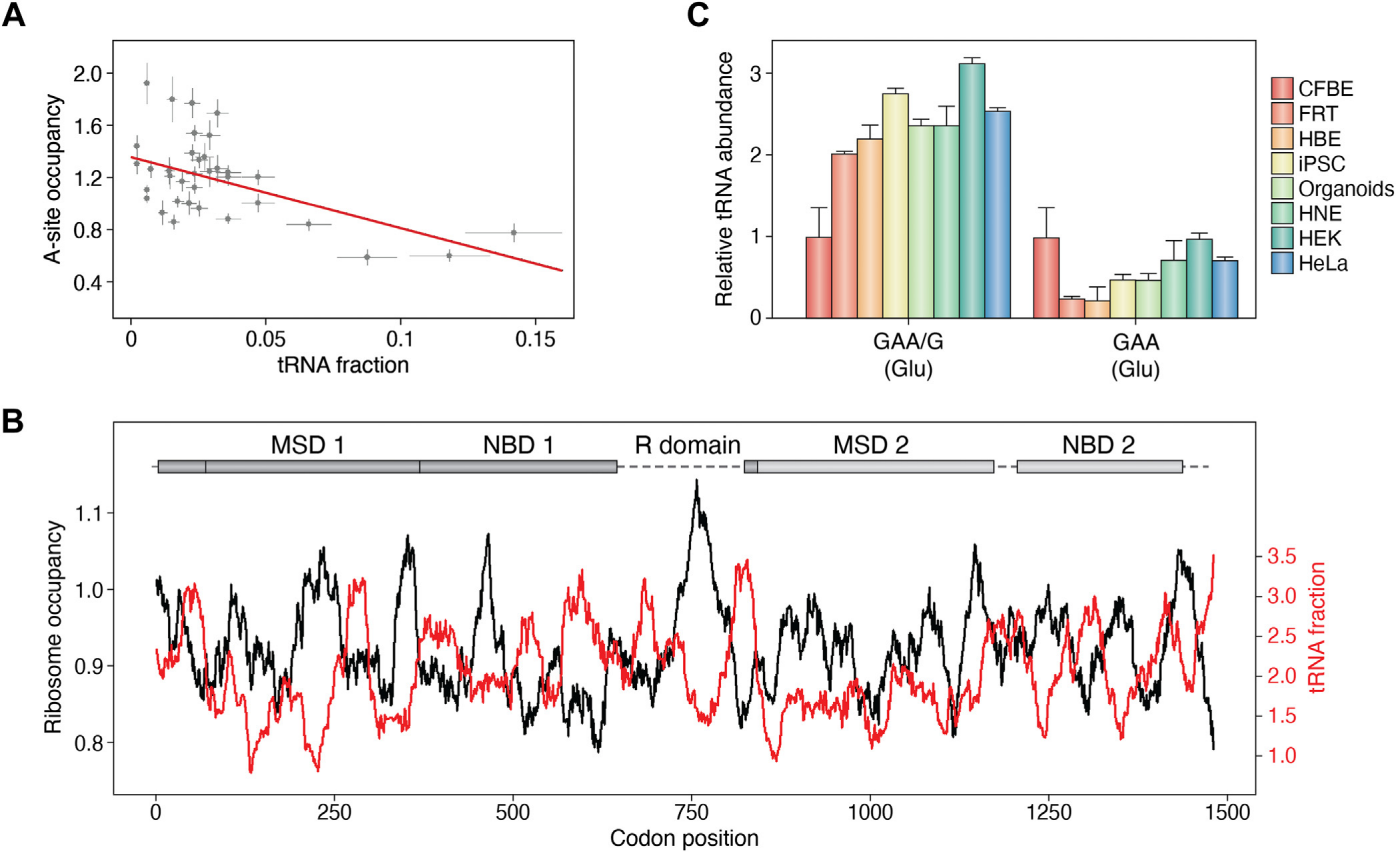
**tRNA abundance determines translation velocity of a codon and can be used to estimate the effect of a mutation.***A*, correlation in CFBE41o^−^ cells between the concentration of tRNA isoacceptors determined by tRNA microarrays (n = 4) and A-site dwelling occupancy from Ribo-seq (n = 3). Data are mean values ± SEM; R = 0.52, Pearson’s correlation coefficient. *B*, the speed of translation (*i.e.,* the ribosomal A-site dwelling occupancy; inferred from Ribo-seq; *black, left axis*) inversely correlates with tRNA concentration profile (*red, right axis*). Example of *CFTR* mRNA translation velocity and tRNA abundance profiles determined by Ribo-seq and tRNA microarrays, respectively, in bronchial epithelial cells CFBE41o^-^. A schematic of CFTR domains is shown at the *top*: MSD, membrane-spanning domain; NBD, nucleotide-binding domain; R, regulatory. *C*, tRNA concentration for both tRNA^Glu^ isoacceptors in various cells and organoids relative to their levels in CFBE41o^−^ cells (which are set at 1). tRNA^Glu^ isoacceptors are depicted with their cognate codon. *From left to right*: CFBE41o^−^ cells; FRT, Fischer rat thyroid cells; HBE, CF patient-derived primary human bronchial epithelia from lung biopsies; HNE, CF patient-derived primary human nasal epithelia from nasal curettage; intestinal organoids; iSPC undifferentiated human induced pluripotent stem cells; HEK 293 cells; and HeLa cells. *Panels A and B* are taken from (132) and *panel C* from (61). CF, cystic fibrosis; CFTR, cystic fibrosis transmembrane conductance regulator.

An alternative approach to determine mutation-driven alterations of translation speed is to quantify the codon translation speed using Ribo-seq. Calibrating the ribosome footprints on the position of the ribosomal A site (the site that accepts the aminoacyl-tRNA) allows for determining the dwelling frequency or ribosome occupancy at A-site codons, which in turn correlates with the translational speed of any given codon (Fig. 5*A*). Overall, the correlation between the A-site codon occupancy and tRNA concentration is reasonably high (Fig. 5*A*), supporting the notion that tRNA concentration could be used as a good predictor of the speed of decoding a specific codon. The power of Ribo-seq can be extended beyond the determination of the global codon speed, and the precise speed of translation of each transcript can be computed. Thus, peaks in ribosome occupancy profiles (*i.e.,* slow-translated regions) are shaped by codons with high-ribosomal A-site codon occupancy and decoded by low-abundance tRNAs (valleys in the tRNA profile), while fast codons decoded by high-abundance tRNAs (peaks in the tRNA concentration profiles) exhibit a low-A-site codon occupancy (Fig. 5*B* and (132)).

A sSNP (c.1584G>A), found in CFTR, reduces expression of CFTR protein in CFBE41o^−^ cells (62). This sSNP changes a Glu GAG codon with a Glu GAA codon. In CFBE41o^−^ cells, the concentration of tRNA_UUC_^Glu^ reading the GAA codon is 2-fold higher than tRNA_CUC_^Glu^ pairing with GAG (61), and an increase of translation velocity of the affected codon is expected. In other cells and tissues, the concentration difference between both tRNA^Glu^ is even higher than in CFBE41o− cells; thus, it is expected that the sSNP-induced codon-speed alterations would be even stronger than in CFBE41o^−^ cells (Fig. 5*C*). This approach exemplifies the power of using the tissue-specific tRNA concentration to estimate the effect of sSNP and nonsynonymous SNP on translation velocity of the affected codon.

## Perspectives

tRNAs are more than passive adaptors in translation that deliver the amino acid to the ribosome. With recent advances in experimental approaches, we are beginning to understand the dynamics of tRNA repertoires and their power in fine-tuning mRNA translation for optimal protein yields and activity in a cell- and tissue-specific manner. The examples of pathologic mutation-induced alteration of codon speed suggest tRNA supplementation as a viable therapeutic option to treat currently incurable human conditions. Indeed, transgenic expression of the most abundant tRNA^Gly^(GCC) isoacceptor alleviated the tRNA^Gly^ sequestration by CMT-GlyRS and rescued protein synthesis and peripheral neuropathy in *Drosophila* and mouse CMT models (165). In cases for which a pathogenic mutation depletes a tRNA (Fig. 3, *B* and *C*), tRNA supplementation could be a viable treatment strategy. Arguably, such a treatment would not be suitable for mutations affecting a single protein-coding gene (Fig. 3*A*), where mRNA replacement (185) or gene editing (186) might be better options.

Spurred by the remarkable breakthrough of the two COVID-19 mRNA vaccines (mRNA-1273 by Moderna and BNT162b2 by BioNTech/Pfizer), many innovative RNA-based treatments (including tRNA-based treatments) may reach clinical translation (see also review by T. Anastassiadis and C. Kohrer in this special issue). However, several critical issues must be solved to unleash the full potential of tRNA therapeutics. Along with problems related to the *in vivo* delivery and the ability to cross multiple biological barriers for access to relevant tissues or cell types, challenges specific for tRNA-based therapies (*e.g.,* tRNA stability, function in translation, and immunogenicity) have to be addressed. Since the expression of the tRNA pools is coordinated and tissue-specific, any dysregulation of the expression of all native isoacceptors from the same family or unrelated tRNA isoacceptor families should be carefully assessed. Upregulation of a single tRNA isoacceptor may reprogram cells toward more proliferative and cancerous phenotypes, as observed for initiator tRNA^Met^ which drives the translational reprogramming of human breast epithelial cells toward proliferative status (187). For natural tRNAs, the therapeutic window might be very narrow and different for each pathology, thus necessitating precise dosing of the tRNA medicines.

The increased application of genome sequencing in diagnostics reveals a plethora of mutations within the same disease-associated gene and in other unrelated genes. The task now is to ascertain the interplay among mutations and, if possible, to estimate their combined effect on the molecular pathology of diseases. Clearly, reconstituting experimentally each mutation or various combinations of mutations cannot match the speed of sequencing-based diagnostics, thus enhancing the value of predictive approaches to better correlate genotypes to phenotypes.

Broadly, discontinuous mRNA translation—which fine-tunes expression, folding, and function of the encoded protein—is determined by the tRNA repertoires in a tissue- and cell-specific manner. Consequently, mutations that alter tRNA availability (Fig. 3) are likely to modulate disease severity and/or be even causal for pathology. The tRNA concentration can be used as a criterion for selecting new variations with phenotypic impact. In particular, this approach holds promise for sSNPs, which are among the most difficult to evaluate and verify experimentally. Although the majority of the sSNPs will remain noncausal for a disease, they may exert epistatic effects on disease-associated mutations and modulate disease severity and progression. Deep quantitative knowledge of tRNA biology in different tissues will allow more accurate understanding of the tissue-specific aspects of pathology. In the not very distant future, spurred by the advances in high-throughput technologies, the tRNAomes could be established as a new line in diagnostics for determining genotype–phenotype association of mutations and individual response to personal medicine treatments.

## Conflict of interest

The authors declare that they have no conflicts of interest with the contents of this article.
